# Supplementary material for: Inferring Latent States and Refining Force Estimates via Hierarchical Dirichlet Process Modeling in Single Particle Tracking Experiments
Source: PLoS One. 2015 Sep 18;10(9):e0137633. doi: 10.1371/journal.pone.0137633 (PMC4575198; doi:10.1371/journal.pone.0137633)
Supplement: S1 Text — (PDF) [file pone.0137633.s001.pdf]

S1 Text: Practical Tips for Computing Data-Driven Priors

Customized or open-source software can be leveraged to calibrate the priors. To illustrate how one can utilize open-source software, we used the initial prior specifications described in Text S5 (note: the initial prior value over R was discussed in the first paragraph of the Results section) to obtain a first pass rough temporal segmentation of the data using the HDP-SLDS technique. After we obtained this segmentation (along with the state parameter estimates), the 1D software provided with Ref. [1] (motion blur parameter set to zero) was used to refine the thermal and measurement noise parameters via MLE. Note that a single MLE parameter estimate was run for each trajectory segment where a state change was determined by the first HDP-SLDS segmentation using this data-driven prior (different segments were not "pooled" to get a single MLE; each segment was used to compute a MLE vector). Subsequently, we used the HDP-SLDS estimates of $\vec{\mu}$ and F (coming from the posterior median) along with the mean of the aforementioned thermal and measurement noise estimates to provide initial guesses to the parameters $\vec{A}$, B and C (see Text S3 for mapping). These initial parameters were then used to seed an MLE search of the 2D overdamped Langevin model described in Ref. [2]. Finally, the component-wise median of the MLE parameter vectors computed along a single trajectory (computed for each segment identified in the first round of the HDP-SLDS analysis) was used to derive the "data-driven priors" used by a second HDP-SLDS processing step (note: only trajectory segments with greater than 100 consecutive temporal observations were analyzed via MLE).

Alternatively, the original procedure outlined in Ref. [2] could be leveraged to more directly provide "data-driven priors" via an initial ad hoc windowed local MLE approach (e.g., reducing the number of HDP-SLDS passes from two to one). The main advantages of the approach outlined above is: (i) more open-source code can be leveraged in the computation; (ii) the HDP-SLDS technique can be used for segmenting and estimating initial MLE parameters in scenarios where parameters may change abruptly (e.g., the use of a SLDS structure permits one to handle a variety of globally non-linear dynamics); and (iii) if reliable subjective information is known, a researcher can leverage this information to seed the initial HDP-SLDS estimator. However, it is advised to check consistency of the data to the model should still be tested after the final state and parameter estimates are computed via formal hypothesis testing [2].

References

1. Michalet X, Berglund A (2012) Optimal Diffusion Coefficient Estimation in Single-Particle Tracking. Phys Rev E 85: 061916.

2. Calderon CP, Thompson MA, Casolari JM, Paffenroth RC, Moerner WE (2013) Quantifying Transient 3D Dynamical Phenomena of Single mRNA Particles in Live Yeast Cell Measurements. J Phys Chem B 117: 15701–13.
