# Supplementary material for: Inferring Latent States and Refining Force Estimates via Hierarchical Dirichlet Process Modeling in Single Particle Tracking Experiments
Source: PLoS One. 2015 Sep 18;10(9):e0137633. doi: 10.1371/journal.pone.0137633 (PMC4575198; doi:10.1371/journal.pone.0137633)
Supplement: S2 Text — (PDF) [file pone.0137633.s002.pdf]

S2 Text: Control Simulation Results

Figure S1 displays a simulated 2D trajectory with multiple state changes. The known states as well as a representative draw from the posterior state assignments are displayed. The HDP-SLDS segmentation was able to identify subtle changes in the dynamics when the measurement noise prior was precisely prescribed. The average normalized Hamming distance (i.e., the number of incorrect state assignments divided by the total number of time series entries where a Hamming distance of 0 indicates a perfect match and a distance of 1 indicates no correct assignments) measured in the posterior when the exact prior was used for segmentation was 0.2285 for this trajectory. Contrast this to the average Hamming distance of 0.4068 obtained when the prior for R was misspecified. Here, the true measurement noise was $40nm$ for both X and Y, but the misspecified prior assumed a measurement noise prior mean of $20nm$. Although this is just one trajectory, results shown later and in Ref. [1] illustrate that an improperly tuned R can adversely affect state segmentation. In what follows, the phrase "Hamming distance" will refer to the average normalized Hamming distance computed using the known truth and the HDP-SLDS output.

Note that the vbSPT method of Ref. [2] was only able to infer 2 states even if we allowed the model to consider a model using the correct number of distinct dynamical states (7 in this simulation). However, the comparison is not entirely fair since many of the state changes are induced by changes in F and $\vec{\mu}$. The current version of vbSPT does not account for variations induced by changing force fields or inhomogeneous diffusion coefficients; the vbSPT method also ignores the statistical effects of measurement noise (accurately modeling this noise is shown here and in Ref. [1] to be important to HDP-SLDS's segmentation accuracy by large scale simulations). This example is shown to illustrate the importance of a "properly-tuned" prior in an HDP-SLDS analysis aiming at state segmentation. The example also shows the accuracy one can obtain if position dependent force fields and measurement noise are properly accounted for by the underlying dynamical models driving the likelihood function.

In Fig. S2, we show results from a batch of multiple simulations where trajectories were simulated to precisely match the conditions assumed by the HDP-SLDS. The resulting data was then analyzed using the HDP-SLDS segmentation. For a baseline, we computed the median Hamming distance (computed over 10^4 MCMC samples) for a situation where the priors, model and data generating process were all in perfect agreement (i.e., a highly idealized situation); the model and sampling parameters are provided in Text S5. The histogram of the aforementioned median distances extracted from the posterior of 1000 independent simulated trajectories are labeled as "Reference Hamming Distance" in the bottom panel of Fig. S2 (note: each of the 1000 trajectories were analyzed by 10^4 MCMC iterations and the HDP-SLDS performance for each trajectory was summarized by the median Hamming distance observed over the MCMC). We then reanalyzed the same set of trajectories by tweaking two different HDP-SLDS parameters

(the same trajectory realizations were reanalyzed to avoid variability in results induced by different random number streams). First we modified the hyperparameter determining the mean of R (the baseline had a mean R which was a diagonal matrix having a standard deviation of $40nm$ for both entries and the modified parameters was again diagonal, but had a standard deviation of $20nm$ for both components). The top panel shows the Hamming distance computed under this new prior condition on the y-axis. In this scatterplot, the $x-$coordinate corresponds to the Reference Hamming distance computed and the $y-$coordinate displays the Hamming distance computed with the same underlying trajectories, but the "bad prior" (each scatterplot point corresponds to a different trajectory). This plot quantifies how a subtle misspecification of R can substantially degrade the performance of the HDP-SLDS segmentation. Note that the next set of results demonstrates how even in the long trajectory setting (i.e., large data), artifacts induced by "bad prior" cannot be readily corrected for by the evidence in observational data alone.

The red circles in Fig. S2 correspond to a correctly specified R, but altering the HDP-SLDS sampling parameter K from 10 to 40. Although the HDP-SLDS allows the number of states, K, to tend to infinity, Fox et al. use a weak limit approximation which imposes an upper-bound on the number of permissible states [3]. In the baseline, K was set to 10, which was greater than the number of unique states observed in the simulated trajectories. The typical recommendation for selecting this parameter in HDP-SLDS modeling is to make K larger than the number of expected states. Inflating K to 40 did not result in a systematic worsening of the results relative to the baseline, though increasing the parameter did increase the variability of the computed Hamming distances (i.e., K does not induce "bias" into the computed Hamming distance whereas the base measure parameter R does in this example, but the spread about the mean increased). For users unsure on the number of statistically distinguishable latent states contained in the data of interest, the K result displayed is encouraging, but the base measure sensitivity can be problematic.

Next we probe the sensitivity of hyperparameters determining R further by showing results obtained when studying a long 2D trajectory (see top panel of Fig. S3). When the prior precisely matches the R used in the simulation, the temporal state segmentation is nearly perfect (the median Hamming distance inferred is close to zero). However, when two heuristics are used for specifying R, the Hamming distance jumps to over 0.33 (i.e., there are three statistically distinct states which persist for a long time, but the poorly tuned prior cases only identify the first two states). The first heuristic is from Fox et al. (see Appendix of Ref. [3]); the second heuristic mimics a prior which a SPT subject matter expert might provide; for this heuristic prior, we assumed a mean effective measurement noise standard deviation of $20nm$ and we label this case as "SPT Heuristic Prior" in the plots (note that in many SPT applications, measurement noise is under-estimated or ignored all together [4, 5]). The heuristic of Fox et al. (labeled

as "Fox et al. Heuristic Prior") encounters problems since SPT data often contains large thermal and measurement noise, but the data was subjected to (known and simulated) measurement noise having a standard deviation of $40nm$. Note the new prior labels use all other default settings discussed in Text S5.

Although there is a wide body of literature [6,7] for computing theoretical lower bounds for localization noise limits (a major contribution to the effective measurement noise, R), the actual measurement noise often deviates substantially from these computable bounds due to a variety of factors which are often unavoidable when one studies *in vivo* motion [4,5]. Fortunately, a poorly specified prior is readily identified by goodness-of-fit hypothesis testing [5]. For example, when using the "SPT Heuristic Prior" discussed above, the misspecification of R leads to rejection of the model inferred directly from the HDP-SLDS method even when the conservative Bonferroni correction [8] is used to adjust for multiple testing (p-values $< 1 \times 10^{-12}$ were observed; see Results section for hypothesis testing information). Using the "Fox et al. Heuristic Prior" to compute the prior mean of R (see Appendix of Ref. [3]) leads to even larger rejection when plugging in the HDP-SLDS parameter estimates obtained (p-values less than machine double precision are computed) since the measurement noise is even further off in this case. For example, the true value for the simulation measurement noise is $40nm$ for each component, but the heuristic of Ref. [3] results in a posterior mode of $222.14nm$ for the Y component of the localization precision. This biased measurement noise estimate is the main cause for the small p-value (note also that this over estimate of a component of R also degrades SPT-SLDS segmentation). When the parameter estimates, $\hat{\theta}$, are obtained using the correct prior (i.e., a prior computed using the known mean of the data generating process), no rejection occurs and the diffusion coefficients and other kinetic parameters are consistently estimated. The results obtained when one divides the original time series shown in Fig. S3 into six uniformly spaced windows and applies the scheme discussed in the first paragraph of Text S1 are displayed in Fig. S4.

In Fig. S5, we confirm that "sticky parameter" [3] can be inferred from the data even if state transitions are short lived and highly transient. The spikes in the X time series (green) are meant to mimic "background flashes" in the fluorescent signal. These flashes were introduced periodically at every 100th observation. The HDP-SLDS analysis outlined in Text S1 was still able to create a new state for this periodically occurring signal while retaining high accuracy in the segmentation.

References

1. Calderon CP (2014) Data-Driven Techniques for Detecting Dynamical State Changes in Noisily Measured 3D Single-Molecule Trajectories. Molecules 19: 18381–18398.

2. Persson F, Lindén M, Unoson C, Elf J (2013) Extracting Intracellular Diffusive States and Transition Rates from Single-Molecule Tracking Data. Nat Methods 10: 265–271.

3. Fox E, Sudderth EB, Jordan MI, Willsky AS (2011) Bayesian Nonparametric Inference of Switching Dynamic Linear Models. IEEE Trans Signal Process 59: 1569–1585.

4. Berglund AJ (2010) Statistics of Camera-Based Single-Particle Tracking. Phys Rev E 82: 011917.

5. Calderon CP, Thompson MA, Casolari JM, Paffenroth RC, Moerner WE (2013) Quantifying Transient 3D Dynamical Phenomena of Single mRNA Particles in Live Yeast Cell Measurements. J Phys Chem B 117: 15701–13.

6. Ober RJ, Ram S, Ward ES (2004) Localization accuracy in single-molecule microscopy. Biophys J 86: 1185–200.

7. Smith CS, Joseph N, Rieger B, Lidke KA (2010) Fast, single-molecule localization that achieves theoretically minimum uncertainty. Nat Methods 7: 373–5.

8. Efron B (2010) Large-Scale Inference: Empirical Bayes Methods for Estimation, Testing, and Prediction. Cambridge: Cambridge University Press.
