# Supplementary material for: Inferring Latent States and Refining Force Estimates via Hierarchical Dirichlet Process Modeling in Single Particle Tracking Experiments
Source: PLoS One. 2015 Sep 18;10(9):e0137633. doi: 10.1371/journal.pone.0137633 (PMC4575198; doi:10.1371/journal.pone.0137633)
Supplement: S3 Text — (PDF) [file pone.0137633.s003.pdf]

S3 Text: Connecting Linear Continuous Time SDE Model Parameters to Those of Discrete SLDS Models

The continuous time analog of Eqn. 1 is given by the following stochastic differential equation (SDE):

$$d\vec{r}_t = \Phi \vec{F}(\vec{r}_t)dt + \sqrt{2}\sigma dB_t. \tag{5}$$

In the equation above, $\vec{F}(\vec{r})$ represents the effective force experienced by a molecule located at position R, Φ models the friction matrix, and σ is related to the diffusion coefficient [1]. This overdamped Langevin framework is fairly general, e.g. non-linear and/or time dependent forces can fit to data using this type of model [1–4]. Note that in the HDP-SLDS, we use F to denote a fixed matrix, whereas in the overdamped Langevin equation above, $\vec{F}(\vec{r})$ is a vector depending on the instantaneous molecular position. In the specific linear parametric models considered in this article, each SDE contributing to an SLDS state (or "mode") is parameterized by a finite dimensional vector denoted by θ. The parameters contained in θ and the remaining terms in Eqn. 5 are defined by the following equations:

$$\vec{F}(\vec{r}) = \vec{A} + B\vec{r} \tag{6}$$

$$\sigma = C \tag{7}$$

$$\Phi = \sigma\sigma^{\mathrm{T}}/k_B T. \tag{8}$$

In the expressions above, $k_B T$ represents Boltzmann's constant multiplied by the system temperature. The net collection of parameters to be estimated is $\theta \equiv (\vec{A}, B, C, R)$; a separate θ is estimated for each unique SLDS state. In the models considered throughout this article, $\vec{A}$ is a 2D vector (i.e., $\vec{A} \in \mathbb{R}^2$). B, C, and R are 2×2 real matrices. The local diffusion coefficient, D, is defined by $D \equiv C \times C^{\mathrm{T}}$. Physical interpretations of the other continuous time parameters are presented in Sec. 1.1.1 of the main manuscript and Ref. [1].

Here we focus on how to map parameters estimated by the HDP-SLDS inference to the continuous time SDE parameters (e.g., this is useful for when wants to compute overdamped Langevin type parameters from the HDP-SLDS output). To simply notation, we will work with the following auxiliary quantities: $\mathcal{Q} \equiv 2C \times C^{\mathrm{T}}$, $\vec{A}' \equiv \frac{1}{2k_B T}\mathcal{Q}\vec{A}$, $B' \equiv \frac{1}{2k_B T}\mathcal{Q}B$; the first auxiliary variable is the instantaneous covariance associated with the continuous time model; the next two auxiliary variables expresses the drift terms of a linear velocity field.

With the auxiliary variables defined above, we show how to transform some of the output of the discrete HDP-SLDS inference algorithm computed using observations evenly spaced Δt time units apart $(\vec{\mu}, F, \Sigma)$ into the corresponding SDE parameters ($\vec{A}'$,B',$\mathcal{Q}$). No transformation is required for R since

observations are discrete in both models. The easiest quantity to extract is $B' = \frac{1}{\Delta t}\log_m(F)$ where $\log_m(\cdot)$ is the principal matrix logarithm (i.e., the inverse of matrix exponential). Given B' and the rest of the HDP-SLDS output, one can readily compute $\vec{A}' = \big(-(Id-F)\times(B')^{-1}\big)^{-1}\vec{\mu}$ where Id denotes the identity matrix; this relation is another simple consequence of the known solution to the linear SDE [5]. Typically, one is given continuous time parameters Δt, $\mathcal{Q}$ and B' and computes the corresponding quantities defining a discrete observation scenario. That is, one quickly computes $F = exp(\Delta t B')$ and uses this to setup the following matrix equation:

$$B'\Sigma + \Sigma(B')^{\mathrm{T}} = F\mathcal{Q}F^{\mathrm{T}} - \mathcal{Q}. \tag{9}$$

One can then solve for Σ using standard control theory tools [6, 7]. However, $\mathcal{Q}$ and hence $C = (\frac{1}{2}\mathcal{Q})^{1/2}$ can be readily solved for explicitly given (F,Σ, B') since the expression above is linear in $\mathcal{Q}$. Recall F and Σ are provided directly by the HDP-SLDS inference and B' can be computed using the procedure described above. Symbolic packages such as Mathematica can be exploited to reliably obtain expressions for $\mathcal{Q}$.

References

1. Calderon CP, Thompson MA, Casolari JM, Paffenroth RC, Moerner WE (2013) Quantifying Transient 3D Dynamical Phenomena of Single mRNA Particles in Live Yeast Cell Measurements. J Phys Chem B 117: 15701–13.

2. Calderon CP, Harris N, Kiang C, Cox D (2009) Quantifying multiscale noise sources in single-molecule time series. J Phys Chem B 113: 138.

3. Calderon CP (2007) On the use of local diffusion models for path ensemble averaging in potential of mean force computations. J Chem Phys 126: 084106.

4. Calderon CP, Martinez J, Carroll R, Sorensen D (2010) P-splines using derivative information. Multiscale Model Simul 8: 1562–1580.

5. Kloeden P, Platen E (1992) Numerical Solution of Stochastic Differential Equations. Berlin: Springer-Verlag.

6. Stengel R (1994) Optimal Control and Estimation. Toronto, Ontario: Dover Publications.

7. Gardiner JD, Wette MR, Laub AJ, Amato JJ, Moler CB (1992) Algorithm 705; a FORTRAN-77 software package for solving the sylvester matrix equation AXBT + CXDT = E. ACM Trans Math Softw : 232-238.
