# Supplementary material for: Inferring Latent States and Refining Force Estimates via Hierarchical Dirichlet Process Modeling in Single Particle Tracking Experiments
Source: PLoS One. 2015 Sep 18;10(9):e0137633. doi: 10.1371/journal.pone.0137633 (PMC4575198; doi:10.1371/journal.pone.0137633)
Supplement: S4 Text — (PDF) [file pone.0137633.s004.pdf]

S4 Text: Dirichlet Distribution and Mixture Models

Dirichlet distributions are multivariate extensions of the beta distribution [2]. The beta distribution's support is the interval $[0, 1]$ and its density is given by:

$$p(x; \alpha_1, \alpha_2) = \frac{\Gamma(\alpha_1 + \alpha_2)}{\Gamma(\alpha_1)\Gamma(\alpha_2)} x^{\alpha_1 - 1}(1 - x)^{\alpha_2 - 1}. \tag{10}$$

The beta distribution is conjugate to the Bernoulli distribution and is commonly used as a prior for the probability of "success" in scenarios where the outcome can be one of two events in Bayesian inference (e.g., heads or tails in coin flipping trials). The Dirichlet distribution generalizes this idea to the scenario where the outcome can be more than two events. For example, the Dirichlet distribution can be used to help infer the probability of each face on a die; one can assume a fair die as a prior (each event has equal probability) and then use the observations of die rolls to infer the probability of each face through the posterior (i.e., use data to determine if the die is actually fair). The density of the more general Dirichlet is shown in Eqn. 11. The Dirichlet distribution is conjugate to the multinomial and it belongs to the exponential family of conjugate priors. The aforementioned feature simplifies posterior computations and has partially contributed to the Dirichlet's popularity in Bayesian analysis [1–3].

A $K-$dimensional random vector $\vec{x}$ is said to be Dirichlet distributed, parameterized by a vector $\vec{\alpha} = (\alpha_1, \ldots, \alpha_K)$ with $\alpha_i > 0 \; \forall \; i$, if its distribution can be written as [2]:

$$p(x; \alpha_1, \alpha_2, \ldots \alpha_K) \sim \text{Dir}(\alpha_1, \ldots \alpha_K) := \frac{\Gamma(\sum_{i=1}^{K} \alpha_i)}{\prod_{i=1}^{K} \Gamma(\alpha_i)} \prod_{i=1}^{K-1} x_i^{\alpha_i - 1}(1 - \sum_{i=1}^{K-1} x_i)^{\alpha_K - 1}. \tag{11}$$

The sum over $K - 1$ components ensures that the sum of the $\vec{x}$'s components is one (hence it is a valid candidate state transition probability in an HMM setting). Assuming a Dirichlet distribution as a prior allows many nice explicit posterior distribution computations (crucial to a full Bayesian analysis) [2], but such priors also allow one to readily formulate "mixture models" [4]. These types of models are relevant because they allow a formal mechanism for assigning θ values to each discrete state, i.e., it provides one a mechanism to assign a dynamical structure to each state. This results in a collection of variables $\{\theta_z\}_{z=1}^{K}$ describing the dynamics and measurement noise statistics of the observed trajectory.

A Dirichlet process results when one allows K to be infinite [1, 3–5]. In Dirichlet process settings, one typically assumes that $\theta_z \sim H(\Theta)$, i.e., the state vector is drawn from a continuous distribution $H(\Theta)$ where Θ is a vector of hyperparameters required to specify the so-called "base measure"H [1, 2]. Even though K is infinite and H comes from a continuous distribution, it can be shown that when one

models each row of a transition matrix as a draw from a Dirichlet process, that each realization contains a countably infinite number of states θ_z with probability one [2, 6]. The transitions characterized by state i are denoted by the countable infinite dimensional transition vector $\vec{\pi}^{(i)}$. The technical problem that arises is that $\vec{\pi}^{(i)}$ and $\vec{\pi}^{(j)}$ have zero probability of having identical θ's associated with their respective (countably infinite) states in a "Dirichlet mixture model" when $i \neq j$ and H is continuous. Hence the probability of returning to a state after exit is zero. This feature complicates using a Dirichlet process in HMM modeling since there is no "sharing" of states between different rows of the transition matrix. The Hierarchical Dirichlet Process (HDP) [3] was designed to overcome this particular problem; the HDP framework allows $\vec{\pi}^{(i)}$ and $\vec{\pi}^{(j)}$ to share a common set of θ's in their components despite H being continuous [3]. The number of states and the transition probabilities can be inferred from the posterior [5]. However, the HDP does not have a mechanism for encouraging state persistence or "self-transitions"; this complicates state identification and estimation in situations where the underlying state remains the same for a relatively long block of time (a common situation in SPT, animal behavior, and target maneuvering [1]). Fox et al. [1] developed the "sticky" HDP-SLDS which adds a hyperparameter designed to promote state persistence through modifying the measures sampled from the HDP. Note that the hyperparameter is learned from the observed time series and the method is surprisingly robust to the assumed initial value of the prescribed "sticky" hyperparameter.

References

1. Fox E, Sudderth EB, Jordan MI, Willsky AS (2011) Bayesian Nonparametric Inference of Switching Dynamic Linear Models. IEEE Trans Signal Process 59: 1569–1585.

2. Ghosh Ramamoorthi, R V, JK (2010) Bayesian Nonparametrics. New York: Springer-Verlag.

3. Teh YW, Jordan MI, Beal MJ, Blei DM (2006) Hierarchical Dirichlet Processes. J Am Stat Assoc 101: 1566–1581.

4. Neal R (2000) Markov chain sampling methods for Dirichlet process mixture models. J Comput Graph Stat 9: 249–265.

5. Fox E, Sudderth E, Jordan M, Willsky A (2010) Bayesian Nonparametric Methods for Learning Markov Switching Processes. IEEE Signal Process Mag 27: 43–54.

6. Sethuraman J (1994) A constructive definition of Dirichlet priors. Stat Sin 4: 639–650.

7. Calderon CP (2013) Correcting for Bias of Molecular Confinement Parameters Induced by Small-Time-Series Sample Sizes in Single-Molecule Trajectories Containing Measurement Noise. Phys Rev E 88: 012707.

8. Fox E, Sudderth E, Jordan MI, Willsky AS (2011) A sticky HDP-HMM with application to speaker diarization. Ann Appl Stat 5: 1020–1056.
