# Supplementary material for: Inferring Latent States and Refining Force Estimates via Hierarchical Dirichlet Process Modeling in Single Particle Tracking Experiments
Source: PLoS One. 2015 Sep 18;10(9):e0137633. doi: 10.1371/journal.pone.0137633 (PMC4575198; doi:10.1371/journal.pone.0137633)
Supplement: S5 Text — (PDF) [file pone.0137633.s005.pdf]

S5 Text: Bayesian Prior Values

To enforce a symmetric positive definite matrix (required for a valid covariance) for both R and Σ, Fox et al. [1] exploited the properties of inverse Wishart prior distributions for these matrix parameters. The inverse Wishart distribution is denoted by $IW(\nu, \Psi)$ where Ψ is a positive definite "scale matrix" and ν is a real number greater than $p-1$ where p is the dimension of Ψ. This distribution is over matrices; if $X \sim IW(\nu, \Psi)$ then $\mathbb{E}[X] = \Psi/(\nu - p - 1)$. This distribution was used by Fox et al. due, in part, to the computational convenience it permits in Bayesian computations.

For R, Ψ was set to $\hat{R} \times (\nu - p - 1)$ where $\hat{R}$ is either a subjective prior or data-driven prior; the nature of the prior for each plot is specified in the Results. Multiplication by the factor $\nu - p - 1$ ensures that the mean of the inverse Wishart is consistent with the specified $\hat{R}$. ν was set to 1000 (this setting was used in the HDP-SLDS toolbox `http://www.stat.washington.edu/~ebfox/software/HDPHMM_HDPSLDS_toolbox.zip`). For the inverse Wishart over Σ, Ψ was selected using both data-driven discrete diffusion coefficient discussed in Text S1 and subjective priors. For the latter, the prior mean Σ_o was used (see last paragraph of this section for the matrix value). Inconsistency between the assumed inverse Wishart prior distribution and data were mitigated by setting $\nu = 10$ (this value allowed substantial dispersion around the Σ prior).

Fox et al. used the so-called matrix normal inverse Wishart prior for the joint prior over F and Σ [1]; this distribution is denoted by $\mathcal{MN}(M, V, L)$ (the marginal posterior of Σ is the standard inverse Wishart discussed above). If $F \sim \mathcal{MN}(M, V, L)$ then $vec(F) \sim \mathcal{N}\big(vec(M), L^{-1} \otimes V\big)$ where $\mathcal{N}(\mu, S)$, is a generic multivariate normal with mean μ and covariance S, $vec(\cdot)$ is the standard vectorization operator (stacking elements of a matrix into a single column) and $\otimes$ denotes the matrix Kronecker product. Hence M specifies the average F drawn in the prior and the variance about this mean value can be adjusted by suitably setting L (a free parameter) for a given Σ_o (see Ref. [1] for the explicit relationship). In our applications, $M = \left(\begin{smallmatrix} 0.9 & 0 \\ 0 & 0.9 \end{smallmatrix}\right)$ and the standard deviation around the diagonal components of M was tuned to 0.15; these values readily handle pure diffusion (i.e., F is the identity matrix and $\vec{\mu}$ is zero) and "confined" diffusion parameter regimes (the priors assumed cover the "confined" regimes studied in Ref. [2]). For the prior over $\vec{\mu}$, a Gaussian with mean zero and a diagonal covariance with $100^2 nm^2$ on each diagonal entry was used (this value was motivated by the observed variation in the chromatid trajectories analyzed).

The parameter K used in "weak limit approximation" [1,3] truncation reduces the infinite state space model to a finite state space (this parameter is denoted by K_z in the HDP-SLDS toolbox mentioned above and denoted by L in Refs. [1,3]). The truncation affords several computational MCMC sampling advantages over other approximations of the infinite dimensional HDP [3]. Recall that Fig. S2 illustrates that this truncation does not introduce systematic bias (hence precise tuning for this parameter is not a

major concern), so we used $K = 10$ as our default setting for this parameter; this value is much higher than the expected number of resolvable states in the experimental SPT trajectories we analyzed here. We also carried out a sensitivity analysis to many of the other default "concentration" hyperparameters and confirmed results stated by the original authors in Ref. [4], namely segmentation results are not substantially affected by the concentration parameters. Hence, the remaining sampling and data generating parameters were left unchanged from the default settings in the publicly available HDP-SLDS toolbox. The simulations studied in this work were generated using these priors to randomly generate data discretely observed every $\Delta t = 0.455s$ (corresponding to the net experimental frame rate of 22 frames/s).

In the large scale simulations cases studied, the parametric forms of the priors and parameter settings not discussed below were identical to those discussed above. Except instead of using data-driven priors, we used the fixed matrices $\Sigma_o = \text{diag}([2.2 \times 10^{-4}, 2.5 \times 10^{-4}])[\mu m^2]$ and $R_o = \text{diag}([40^2, 40^2])[nm^2]$ as the prior means over the discrete process and measurement noise, respectively (recall diag($\cdot$) denotes the square diagonal matrix formed by the arguments). These values were selected by analyzing a large population of chromatid trajectory segments. Dispersion around these states in the HDP simulations were determined by the sampling parameters and prior distributions discussed above. The parameter determining the variance of $\vec{\mu}$ in the DGP was set to $Id \times 200^2 nm^2$ (slightly larger than the assumed prior dispersion). In the HDP-SLDS analyses of the large scale simulation trajectories, all priors matched the data generating process (DGP) unless otherwise explicitly specified in the text.

Note that we also considered putting a "diffuse" prior on the ν for both Σ and R. However, it was observed that doing so substantially increased the variability of the MCMC sampling. Techniques aiming towards putting a better "non-subjective" prior over both Σ and R would be interesting future research directions. We emphasize that the base measure parameters determining the posterior and prior values of R, Σ, F significantly affected segmentation results in the SPT applications studied.

References

1. Fox E, Sudderth EB, Jordan MI, Willsky AS (2011) Bayesian Nonparametric Inference of Switching Dynamic Linear Models. IEEE Trans Signal Process 59: 1569–1585.

2. Calderon CP (2013) Correcting for Bias of Molecular Confinement Parameters Induced by Small-Time-Series Sample Sizes in Single-Molecule Trajectories Containing Measurement Noise. Phys Rev E 88: 012707.

3. Fox E, Sudderth E, Jordan MI, Willsky AS (2011) A sticky HDP-HMM with application to speaker diarization. Ann Appl Stat 5: 1020–1056.

4. Fox E, Sudderth E, Jordan M, Willsky A (2010) Bayesian Nonparametric Methods for Learning Markov Switching Processes. IEEE Signal Process Mag 27: 43–54.
