# Supplementary figures and images for: Inferring Latent States and Refining Force Estimates via Hierarchical Dirichlet Process Modeling in Single Particle Tracking Experiments
Source: PLoS One. 2015 Sep 18;10(9):e0137633. doi: 10.1371/journal.pone.0137633 (PMC4575198; doi:10.1371/journal.pone.0137633)

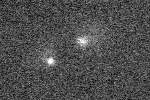

Supplement: S1 Data — (ZIP) [file pone.0137633.s006.zip › Data S1/Fig2_11.27.13 GFP single Stream1 cropped.stk]

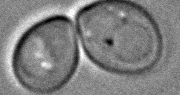

Supplement: S1 Data — (ZIP) [file pone.0137633.s006.zip › Data S1/Fig2_11.27.13 WhiteLightROI_PlotInArticleFigFlippedRelativeToImageJview.tif]

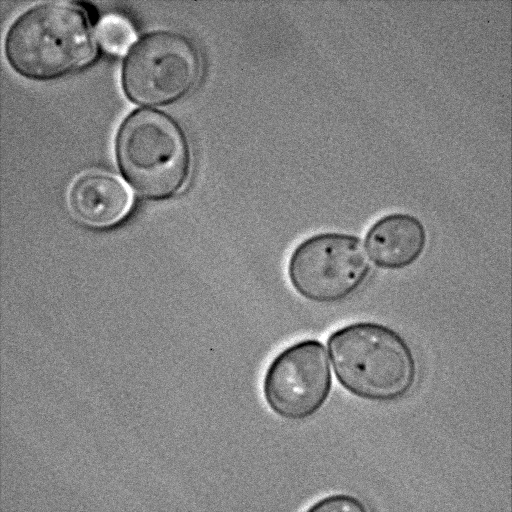

Supplement: S1 Data — (ZIP) [file pone.0137633.s006.zip › Data S1/Fig2_FullView_WhiteLight_PlotInArticleFigFlippedRelativeToImageJview.tif]

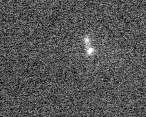

Supplement: S2 Data — (ZIP) [file pone.0137633.s007.zip › Data S2/Fig4_12.4.13 GFP single Stream4 cropped.stk]

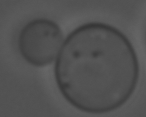

Supplement: S2 Data — (ZIP) [file pone.0137633.s007.zip › Data S2/Fig4_12.4.13 WhiteLight Stream4 cropped.stk]

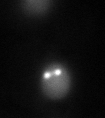

Supplement: S3 Data — (ZIP) [file pone.0137633.s008.zip › Data S3/Fig5_12.3.13 GFP single Stream2 cropped.stk]

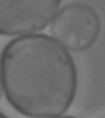

Supplement: S3 Data — (ZIP) [file pone.0137633.s008.zip › Data S3/Fig5_12.3.13 WhiteLight Stream2 cropped.stk]

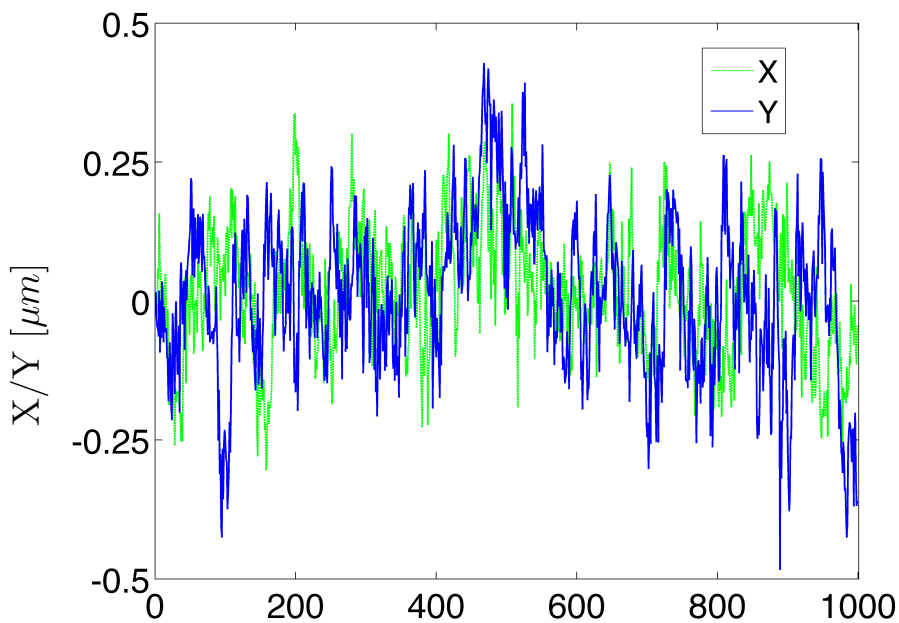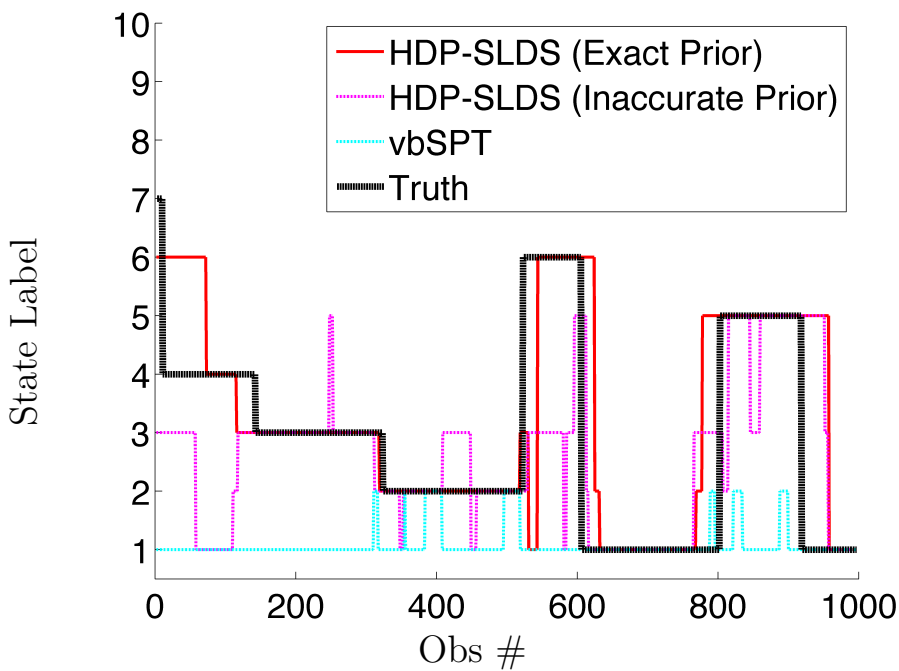

Supplement: S1 Fig — Top panel: Simulated time series exhibiting rapid state transitions. Bottom panel: True state sequence (7 unique states) along with HDP-SLDS and vbSPT estimates of the state obtained using two different priors. The case labeled as “Exact Prior” used a prior matching the data generating process precisely and that labeled as “Inaccurate Prior” misspecified the measurement noise standard deviation by a factor of 0.5 in the corresponding prior parameter. (PDF) [file pone.0137633.s009.pdf]

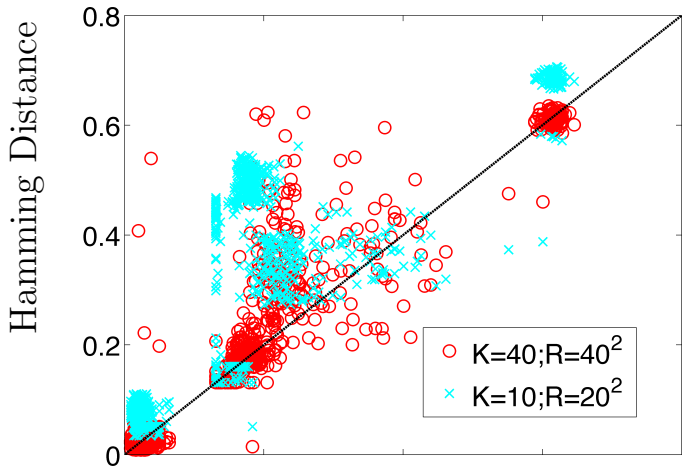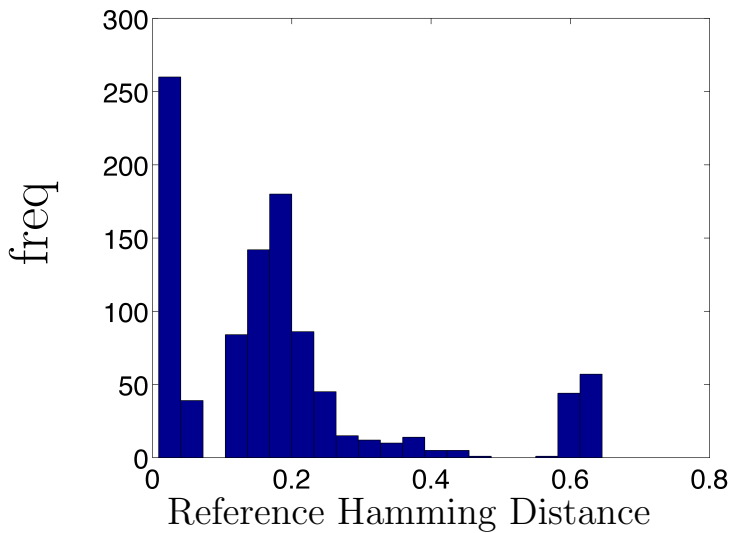

Supplement: S2 Fig — 1000 trajectories (each containing 1000 temporal observations) were simulated. A histogram of the trajectorywise median Hamming distance computed using the HDP-SLDS approach [32] (see S2 Text) observed during MCMC iterations (104 MCMC samples were drawn for each trajectory and for each MCMC sample the Hamming distance was computed), is shown in the bottom panel for the case where the priors precisely matched the data generating process (i.e., “Reference Hamming Distance”). Note that the HDP-SLDS sampling information is collapsed to a single number (the histogram summarizes a collection of the median Hamming distances computed separately for each of the 1000 trajectories). The effects of varying two sampling parameters R and K are shown in the top panel via scatter plots; here, the x− axis coordinate corresponds to the “Reference Hamming Distance” and the y− coordinate displays results obtained using the same trajectory as input data, but using an HDP-SLDS analysis with different sampling parameters to compute the Hamming distance. (PDF) [file pone.0137633.s010.pdf]

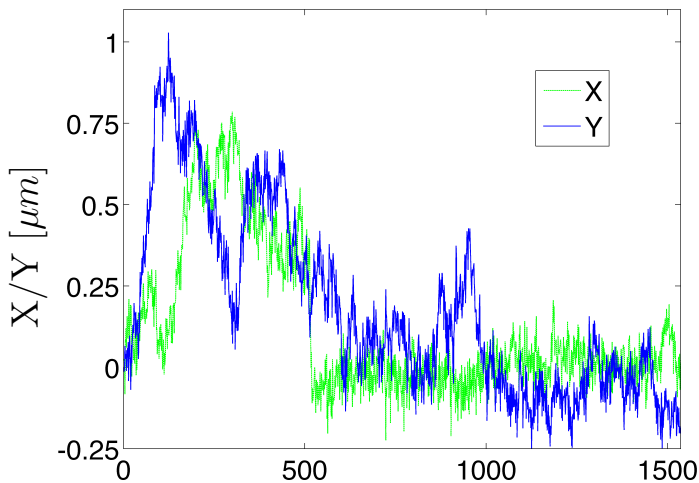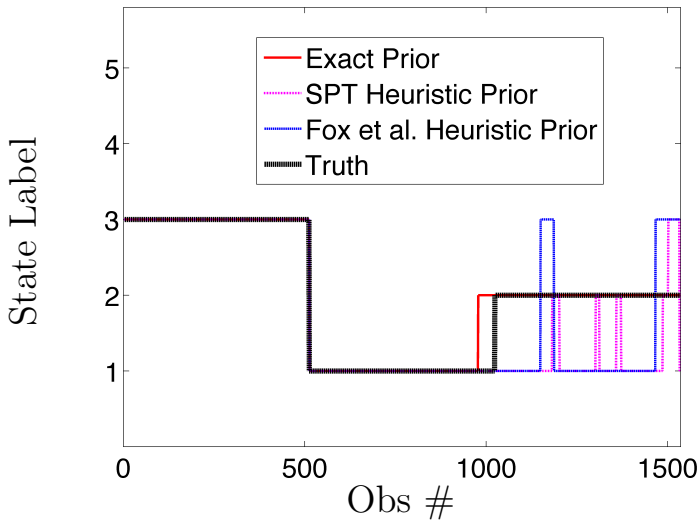

Supplement: S3 Fig — Simulated trajectory where two heuristics fail to identify a long a lived state change near observation 1000. The inaccuracy in the measurement noise in the two heuristic priors is the primary cause for the inability to detect the state change. Note that the case labeled “SPT Heuristic Prior” refers to the situation where the measurement standard deviation was assumed to be half of the true value used in the simulation (see S2 Text for additional details and motivation behind studying these heuristic priors). (PDF) [file pone.0137633.s011.pdf]

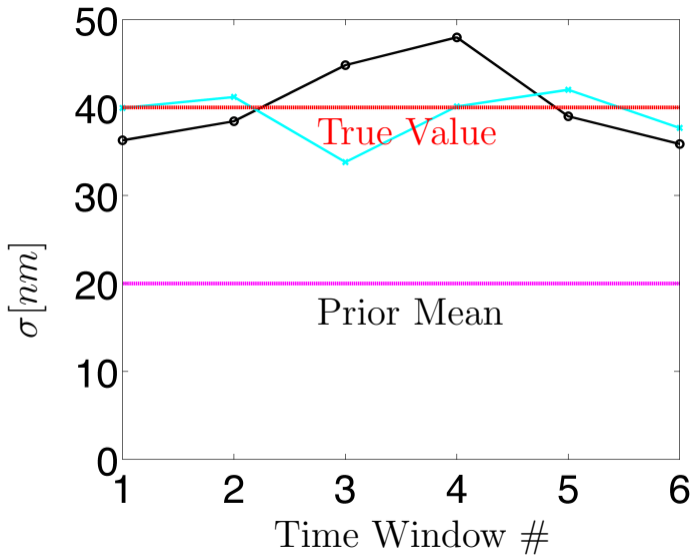

Supplement: S4 Fig — The prior mean of the “SPT Heuristic Prior” (discussed in S2 Text) and true measurement noise of the simulation shown in S2 Fig are denoted by horizontal lines without symbols. The symbol/line plots denote effective noise estimates obtained using a variant (discussed in S2 Text) of the approach advocated in Ref. [23]. The black symbol/line plot correspond to estimates obtained for the X component and the cyan plots to estimates obtained analyzing Y component of the diagonal matrix R. (PDF) [file pone.0137633.s012.pdf]

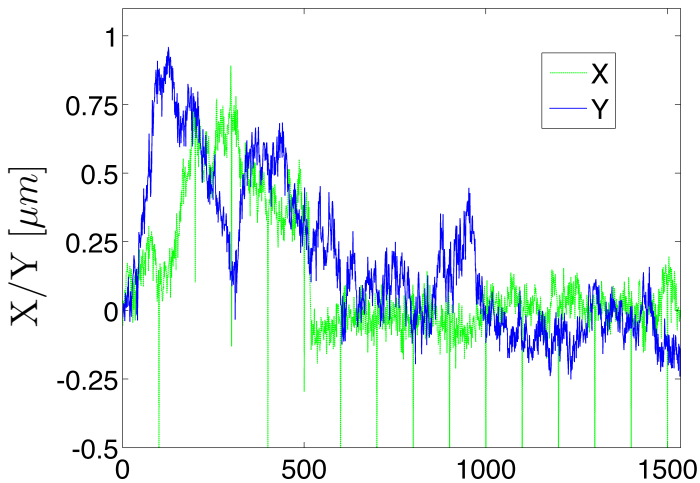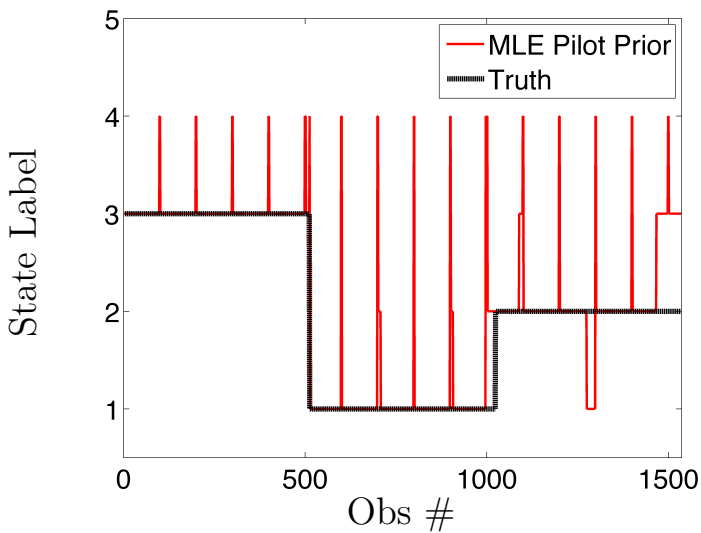

Supplement: S5 Fig — Same trajectory as S3 Fig, however large periodic outliers (meant to mimic fluorescent background flashes), were introduced. Note that the three long lived states are still identified, but a fourth new state corresponding to the simulated background flashes is created. Results labeled “MLE Pilot Prior” in the legend refer to the first data-driven method discussed in S1 Text. The “MLE Pilot Prior” segmentation is indistinguishable from those obtained using the “Exact Prior” (e.g., a prior matching the data generating process without outliers). This result shows that the HDP-SLDS has the ability to detect transient outlier events which can be encountered in SPT analysis. (PDF) [file pone.0137633.s013.pdf]

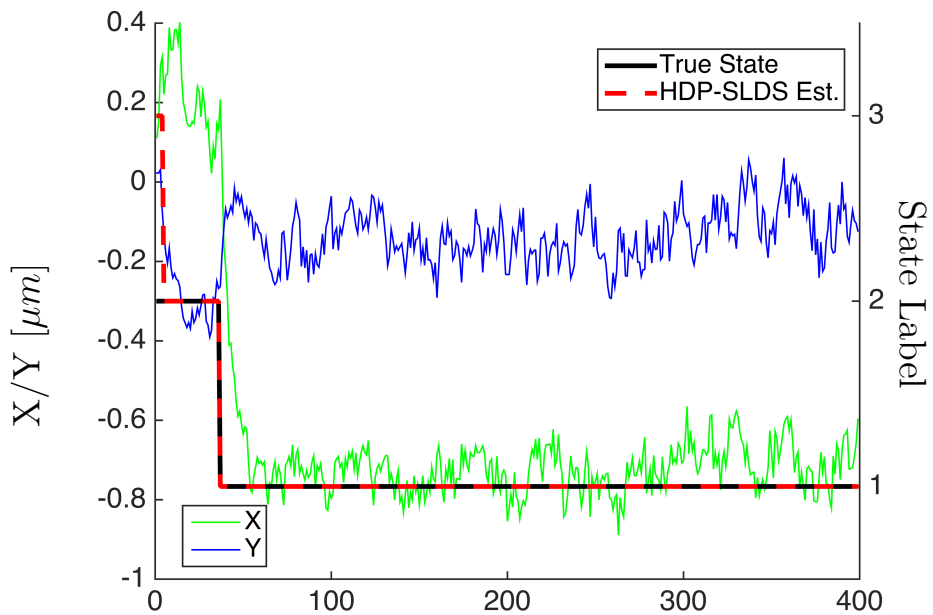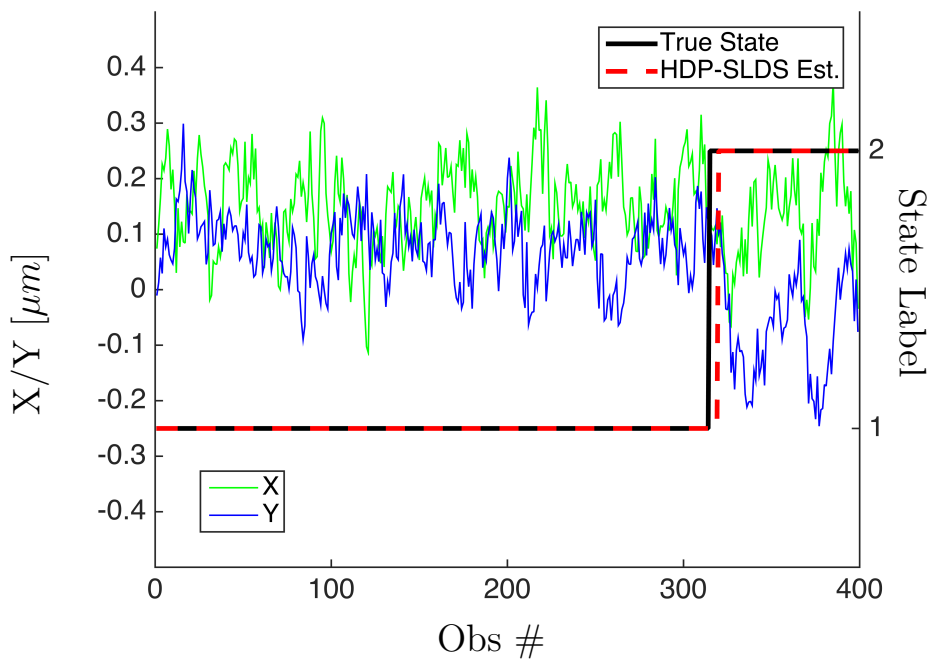

Supplement: S6 Fig — In both pairs of trajectories, a Gaussian measurement noise with a standard deviation ramping linearly from 15nm to 35nm (for both components) over 400 observations was used to generate two pairs of trajectories (the time dependent measurement covariance matrix used was obtained by multiplying the 2D identity matrix by the square of the linearly varying standard deviation). In the HDP-SLDS formulation considered, the trajectories assume a fixed prior measurement noise covariance over the course of the trajectory (the inverse Wishart prior mean for R was the identity matrix multiplied by 252 nm 2). The true state and HDP-SLDS state estimates are shown for one sample of the MCMC iteration. Note that this is a situation where a fundamental assumption of the HDP-SLDS model is violated (i.e., the gradual “continuous” time trend in parameters violates the constant R assumption of the HDP-SLDS model). The top panel shows a trajectory with a visually obvious “mean shift” and the bottom trajectory exhibits a subtler change induced by jumps in the diffusion coefficient and F. These plots illustration that the HDP-SLDS can achieve accurate segmentation for both obvious and subtle changes in parameters despite some of its fundamental modeling assumptions being violated by the observed data. (PDF) [file pone.0137633.s014.pdf]

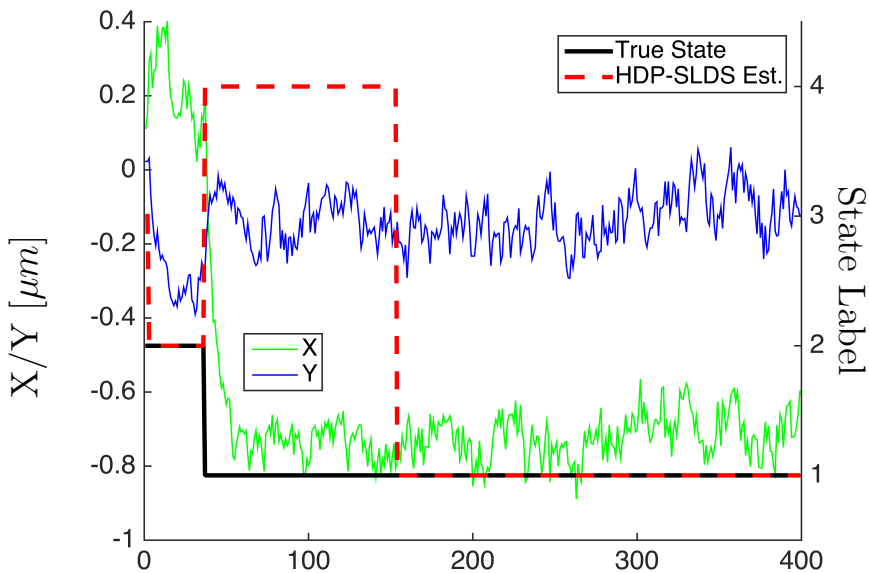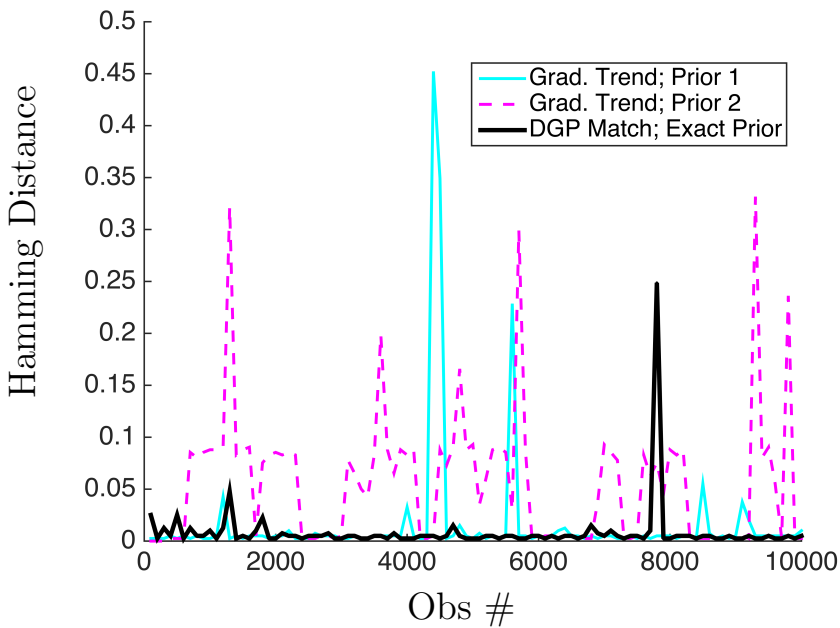

Supplement: S7 Fig — Top Panel: Analysis of the same trajectory in S6 Fig, except the HDP-SLDS routine used the identity matrix multiplied by 152 nm 2 for the mean measurement noise covariance of the inverse Wishart prior. With this prior, one can readily observe artifacts in the HDP-SLDS state estimates induced by the gradual trend in measurement noise. Recall that our HDP-SLDS formulation assumes a constant measurement noise for the duration of a single trajectory so there is model misspecification present (however this analysis uses a “bad prior” in addition to the model misspecification studied in S6 Fig). Bottom Panel: average Hamming distance computed in each MCMC draw for various HDP-SLDS runs. The “Grad. Trend” label refers to runs using the noisy X/Y data corrupted by a measurement noise with a linearly ramped standard deviation; the “Prior 1” label refers to the prior used in S6 Fig and “Prior 2” refers to the prior described above (both cases analyzed the same X/Y data, the only difference in output was induced by the prior mean over R producing different measurements). The case labeled “DGP Match” presented the algorithm with the same X/Y data, but added measurement noise with a fixed covariance (a two-dimensional identity matrix multiplied by 252 nm 2). Even when the HDP-SLDS parameters precisely match the DGP occasional state segmentation errors occur due to the random nature of the MCMC sampler used; note that we only declared a state change was implied by the data if the change occurred (at the same time point) for over 25% of the MCMC samples. (PDF) [file pone.0137633.s015.pdf]
